# Safety assessment: predicting fatality rates in methanol plant incidents

**DOI:** 10.1016/j.heliyon.2022.e11610

**Published:** 2022-11-17

**Authors:** Mohd Aizad Ahmad, Zulkifli Abdul Rashid, Ateyah Awad Alzahrani, Mohanad El-Harbawi

**Affiliations:** a INPRES, School of Chemical Engineering, College of Engineering, Universiti Teknologi MARA, 40450 Shah Alam, Selangor, Malaysia; b Department of Chemical Engineering, King Saud University, Riyadh, 11421, Saudi Arabia

**Keywords:** Methanol plant incidents, Fatality rate prediction, ANFIS

## Abstract

In this article, the prediction of fatality accident rate at methanol (MeOH) plant was studied using different assessment methods. The prediction method was performed and simulated using HYSYS, ALOHA, MARPLOT, and MATLAB software. Recent studies for pressure variation up to 442 bar in MeOH synthesis by carbon dioxide (CO_2_) hydrogenation showed that three times more MeOH was produced than in conventional plants, with 90% CO_2_ conversion and 95% MeOH selectivity. However, safety concerns were noted when MeOH production was operated at pressures above 76–500 bar. Therefore, a safety assessment of the pressures between 76 and 500 bar was performed to predict the fatality rate at the MeOH plant. Adaptive Neuro-Fuzzy Inference System (ANFIS) was compared with a conventional analysis by using the consequence method to predict the fatality rate. First, 26 input parameters were simulated in HYSYS, ALOHA, and MARPLOT software by using the consequence method. Then, the input parameters were reduced to six, namely, pressure, mass, volume, leakage size, wind speed, and wind direction, for prediction using ANFIS tool in MATLAB. This study aimed to highlight the accuracy of the fatality rate prediction by using the ANFIS method. In this manner, accurate prediction of fatality rate for MeOH plant incidents was achieved. The prediction values for the ANFIS method was validated using the standard error of the regression. The percent error measurement obtained the lowest regression of 0.0088 and the lowest percent error of 0.02% for Hydrogen (H_2_) Vapor Cloud Explosion (VCE) ident. Therefore, the ANFIS method was found to be a simpler and alternative prediction method for the fatality rate than the conventional consequence method.

## 1 Introduction

MeOH has vital potential as a fuel because it could be used as a normal fuel for mobile transportation (Valera and Agarwal, 2020) and selected as a feedstock for the production of olefins from which hydrocarbons could be synthesized (Olah, 2005; Olah et al., 2018). The production of MeOH as a liquid fuel has been studied for various modes of transportation, such as shipping (Brynolf et al., 2014) and aviation (Atsonios et al., 2015). Meanwhile, production of H_2_ from MeOH for vehicles was found to have potential (Nielsen et al., 2013). In particular, the pursuit of a “methanol economy” could spur industrialization, as proposed in China and the United States (US) (Pérez-Fortes et al., 2016). However, producing MeOH by using the conventional method, *i.e*., from coal or natural gas, contributes to increased greenhouse gas emissions and increases water consumption (Yao et al., 2018).

Waste CO_2_ could be converted into useful chemicals, such as MeOH, which could be produced in two manners: first is the direct reaction of CO_2_ with H_2_ and second is the conversion of CO_2_ to CO, after which CO could react with H_2_ (Van-Dal and Bouallou, 2012; Ahmad et al., 2021). These two methods could be used to mitigate CO_2_ from the atmosphere. In addition, alternative methods that reduce CO_2_ concentration in the atmosphere include electrochemical reduction of CO_2_ in fuel cells (Albo et al., 2015; Agarwal et al., 2011; Yamamoto et al., 2002) and conversion of CO_2_ to fuels by using a photoelectrochemical approach (Ammpelli et al., 2011). The production of MeOH from CO_2_ is being studied by various researchers by using different analytical methods. These methods may include CO_2_ reduction, economic or financial analysis, and energy analysis (Van-Dal and Bouallou, 2012, 2013; Pérez-Fortes et al., 2016; Tidona et al., 2013).

Various researchers have studied the effect of different parameters on the production of MeOH by using CO_2_ capture, such as pressure (Bansode and Urakawa, 2014; Gaikwad et al., 2016; Gaikwad, 2018), temperature (Gaikwad et al., 2016; Gaikwad, 2018), molar ratio (Bansode et al., 2014; Ghosh et al., 2021; Kanuri et al., 2021), and gas-hourly space velocity (Bansode et al., 2014, Gaikwad et al., 2016; Portha et al., 2017; Gaikwad, 2018; Koh et al., 2021; Ghosh et al., 2021). Processes with low operating conditions, such as temperature and pressure, are well known to be inherently safer than those with extreme conditions (Heikkilä, 1999; Amyotte and Khan, 2021). As a rule, processes with high pressure and high temperature in combination increase the possibility of chemical leakage and thus increase the possibility of accidents, such as explosions and fires (Srinivasan and Nhan, 2008). In the preliminary design phase of a chemical plant, engineers analyzed the possible different pressure conditions of CO_2_-to-MeOH plants. These conditions were subjected to various consequences, with fatalities among the outcomes evaluated. The amount of mixture in the reaction between CO_2_ and H_2_ could result in different densities, volumes, and masses of the mixture. The mixture consists of the following individual chemical components: CO_2_, H_2_, MeOH, CO, and water (H_2_O). The effects of the leakage of such a mixture were determined by various factors, such as pressure, mass, volume, size of the leak, wind direction, and wind speed. These factors must be simulated to estimate the mortality rate. Therefore, simplifying the prediction of fatalities by using advanced prediction models, such as artificial intelligence (AI), is important for the prediction of the fatality rate due to the release of hazardous materials to be easier than before and for quick estimation of the consequences when various inputs need to be enteredadaptive neuro-fuzzy inference system (ANFIS) could be used to quantify the correlation between input and output data (Jang, 1993; Jang et al., 1997). AI prediction method has been widely used in various environmental studies (Zhu et al., 2019; Bhagat et al., 2020; Tiwari et al., 2018). However, to the best of the authors’ knowledge, this technique has not yet been applied in the field of process safety or process risk assessment. refore, the present work aimed to study the prediction of fatality rates in methanol plant accidents by using consequence and ANFIS prediction methods.

## 2 Methodology

**Figure 1:**
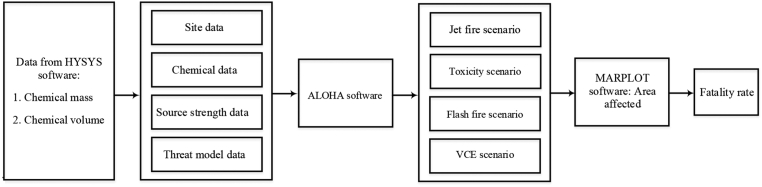
Flow diagram for the consequence prediction method.

**Figure 2:**
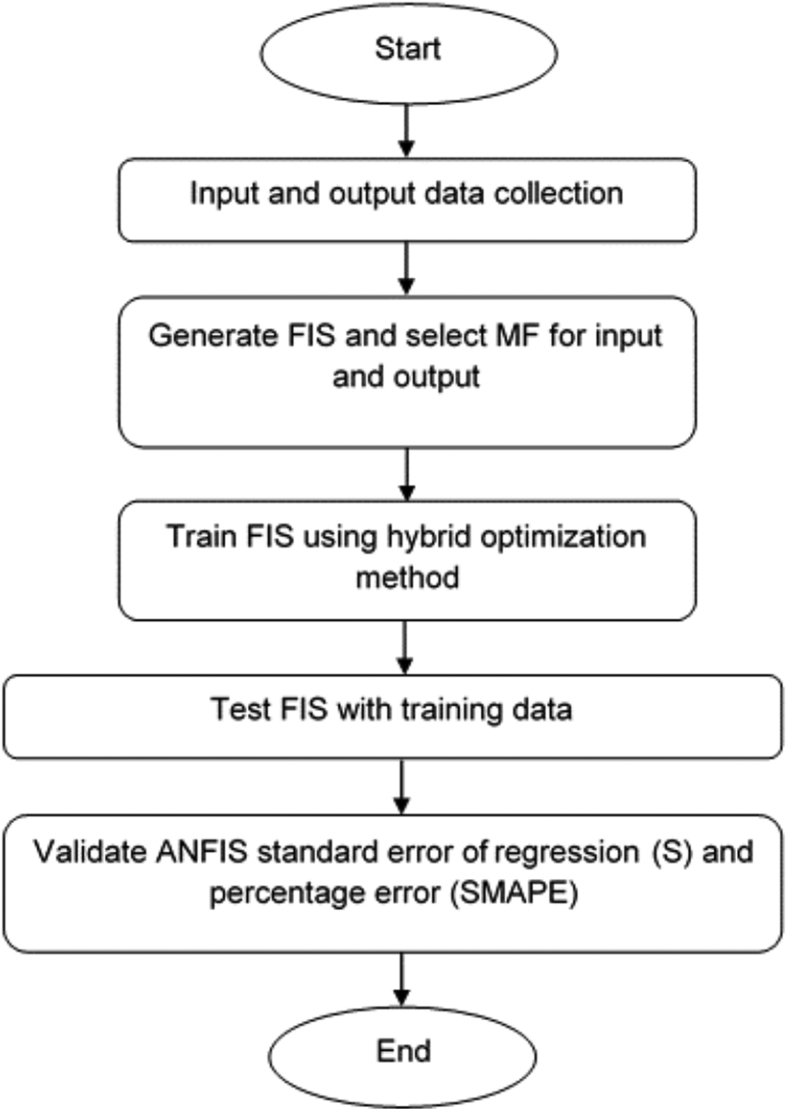
Flow diagram of the ANFIS prediction method.

In this paper, two methods were used to estimate the severity of a hazardous release from reactor systems, namely, the consequence prediction method and the ANFIS prediction method. The ANFIS prediction was developed using two different membership functions, triangular and Gaussian shape. The most important parameter for the severity of the incidents is the number of fatalities in the area of the red danger zone. The severity parameter was simulated using the consequence prediction method. In this method, the mass and volume of hazardous chemicals were obtained from the Hyprotech Systems (HYSYS) version 8.8 software. These data were fed into the software Arial Location Hazardous Atmosphere (ALOHA) version 5.4.7, which contains data on the wind speed and the size of the leakage. Finally, the software Mapping Application for Response, Planning, and Local Operational Tasks (MARPLOT) version 5.1.1 was used to plot the red zone threat footprint on target maps. The input and output variables from the simulation data were then used for prediction in the ANFIS prediction method. Figure 1 presented flow diagram for the consequence prediction method while Figure 2 depicted flow diagram of the ANFIS prediction method.

### 2.1 Consequence prediction method

Various input data were simulated from the severity model by using the simulation software ALOHA. Table 1 shows the input and output variables for the consequence model methodhe variables consist of site, chemical, atmospheric, source strength, and threat model data. Figure 1 shows a flow diagram of the consequence prediction method. The input data could be divided into five categories: site data, chemical data, atmospheric data, source strength model input, and threat model input. These inputs include 24 inputs for toxicity, jet fire, and flash fire incidents, and 26 inputs for vapor cloud explosion (VCE) incident. Site data requires the user to enter the location, building type, building surrounding, date, and time of a possible event. Chemical data instructs the user to select chemicals in the ALOHA database showing characteristics, such as CAS number, toxicity level, lower explosive limit (LEL), upper explosive limit (UEL), boiling point, vapor pressure, and saturation concentration, at ambient conditions. For atmospheric data, users required to enter the wind speed and direction, wind measurement height, ground roughness, cloud-covered sky value, air temperature, atmospheric stability classes, height of inversion, and humidity. However, source strength inputs required data on the orientation of the tank (i.e., whether it is a horizontal or vertical tank with cylinders or spheres). Entering the size of the tank in terms of diameter, length and volume, the state of the chemical, i.e., liquid or gaseous, is also necessary. ALOHA also requires the data of the temperature in the tank, the mass of the gas in the tank or the pressure in the tank; the category of the tank failure; whether chemical burns (jet fire) or not when released into the atmosphere; the shape of the leakage opening, i.e., circular or rectangular; the diameter of the opening; and the choice of whether it is a through hole or a short pipe/valve.

**Table 1:** Input and output variables for the consequence method.

Data Category	Input Variables	Output Variables
Site data	1. Location – location name, approximate elevation, approximate location of the latitude and longitude 2. Building type – enclosed office building/single-story building/double-story building 3. Building surroundings – sheltered surrounding/unsheltered surroundings 4. Date and time – month, day, year, hour and minute	
Chemical data	5. Chemical selected from ALOHA database – Hydrogen, methanol, carbon monoxide, carbon dioxide 6. Chemical characteristic displayed – chemical name, CAS number, molecular weight, toxicity level, LEL, UEL, boiling point, vapor pressure, and saturation concentration at ambient conditions ∗CO_2_ has no LEL and UEL values	
Atmospheric data	7. Wind speed (in meters/second), wind direction (from north, east, south etc.), 8. The height of the wind measurement above the ground 9. Ground roughness – open country/urban or forest/open water 10. Cloud cover – completely overcast/partly overcast/clear 11. Air temperature (in degree Celsius) 12. Stability class – A/B/C/D/E/F 13. Inversion height – no inversion/inversion present (in meters/feet) 14. Humidity – varies from 0 to 100 %	
Source strength data	15. Tank type and orientation – horizontal cylinder/vertical cylinder/sphere 16. Tank size – diameter, length volume (in meters and cubic meters) 17. Chemical state – tanks contain liquid/tank contains gas only/unknown 18. Temperature of chemical within the tank – specify temperature in oC 19. Amount of gas – specify amount in kilogram 20. Type of tank failure – leaking tank, non-burning chemical, burning chemical (jet fire) 21. Shape of the opening from which the pollutant leaks – circular opening/rectangular opening 22. Opening diameter – specify values in meters 23. Leakage through – hole or short pipe/valve	Incident in which chemicals do not burn when released into the atmosphere: Release duration, release rate, total amount release Incident in which chemicals burned as a jet fire: Maximum flame length, burn duration, maximum burn rate, total amount burned
Threat model data	Incident in which the chemical is not burned when released into the atmosphere; Divided into toxic or flash fire or VCE scenario: - If toxicity hazard is selected: -24 (toxic). Input red, orange, and yellow zones for toxicity level of concern. If flash fire hazard is selected: -24 (flash fire). Input red, orange, and yellow zones for flammable level of concern If vapor cloud explosion is selected: -24 (VCE). Vapor cloud ignition's time – unknown/known 25 (VCE). Vapor cloud ignition type – ignition by spark or flame/ignition by detonation 26 (VCE). Level of congestion – congested/uncongested Input red, orange, and yellow threat zones for the overpressure level of concern Incident for burning chemical; Only jet fire scenario: -24 (jet fire). Input red, orange, and yellow threat zones for the thermal radiation LOC--Para Run-on-->	Incident in which chemicals do not burn when released into the atmosphere: Toxicity hazard –vapor cloud area for toxic and distance Flash fire hazard –vapor cloud area for flammable and distance Vapor cloud explosion hazard –vapor cloud area for blast explosion and distance Incident for burning chemical (jet fire): -Thermal radiation hazard – thermal radiation area of jet fire and distance

The results of inputting site, chemical, atmosphere, and source strength data determine the duration of the release, the release rate, and the amount of total release as output when the user selects tank failure, i.e., a chemical that does not burn when it escapes into the atmosphere. The heavy gas model was used for gasses with a density higher than that of air. The outputs were then used by ALOHA to simulate areas vapor cloud areas for toxic, flammable, and blast explosions. However, when tank failures occur, where the chemical burns like a jet fire, the output variables are the maximum flame length, burn duration, maximum burn rate, and total amount burned. The output from this incident was used to estimate the radiant heat area of the jet fire. The input variables for the threat model outputs originated from two categories of tank failures; chemicals that are released into the atmosphere without burning and chemicals that burn immediately when released into the atmosphere and form a jet fire.

Three possible incidents occur for the first type of failure: toxic release, flash fire, and explosion. The first incident simulates the vapor cloud area. For toxic release, the user is required to specify the toxic hazard level of concern (LOC) for the selected chemicals. The LOC of this toxic determines the threat zone for the selected chemical, whether it is indicated as a red, orange, or yellow threat. For the second incident where a flash fire could occur, users could indicate a red threat zone of 60% LEL and 10% LEL as a yellow threat zone to indicate the local area of the flame. If users want to simulate blast area VCE incident, they must enter the time of vapor cloud ignition, regardless of whether or not the time of ignition is known. The type of ignition for the vapor cloud scenario must also be specified, whether it is ignition by sparks or flames or ignition by detonation. In addition, the level of congestion, whether congested or not, must be confirmed. The overpressure level of concern must be identified to classify which parts are within the red, orange, and yellow threat zones. All of these parameters were referred to as inputs to the threat model, which consisted of a heavy gas model for air dispersion, a vapor cloud model for the flammable area, and the overpressure from the VCE model. The results of the source strength model combined with the inputs from the threat model used to run the heavy gas model produced a hazard area called the affected area. The results of the affected area were then used to calculate the mortality rate in the red threat zone.

In this study, a MeOH plant operating at different pressures of 76, 100, 150, 200, 250, 300, 350, 400, 450, and 500 bar was selected as a case study. The pressure of 76 bar was chosen as the minimum pressure on the basis of the typical operating pressure of a commercial MeOH plant, which was also simulated by Perez-Fortes et al. (2016). The high pressure simulated up to 500 bar was intended to mimic the experimental work of Institut Catala dInvestigacio Quimica researchers, who applied pressures up to 442 bar to achieve high MeOH production (Bansode, 2014; Bansode and Urakawa, 2014; Gaikwad et al., 2016; Gaikwad, 2018). The pressure variations were simulated with the HYSYS software, applying the thermodynamic equilibrium of the mixture and using a reactor volume of 7.6 m^3^. Considering the operating temperature was 288 °C, the density of the mixture was generated in accordance with each operating pressure for the value of 288 °C, and the mass of the mixture was calculated by multiplying this density by the reactor volume. The hazardous chemical components in the mixture consisted of CO_2_, H_2_, CO, and MeOH, whose masses were calculated on the basis of their mass fraction multiplied by the mass of the mixture. Meanwhile, the volume for each chemical component in the reactor could be determined by the product of the volume fraction multiplied by the volume of the reactor, 7.6 m^3^. Thus, the mass and volume of CO_2_, H_2_, CO, and MeOH varied at each operating pressure from 76 bar to 500 bar, affecting the mass release and total amount burned for the toxicity and jet-fire scenario and consequently producing a different red zone area footprint. Leaks were assumed to occur through various orifice sizes: small, medium, and large, i.e., 10, 25, and 160 mm, respectively. These representative hole leaks were based on Purple Book guidelines (Stoffen, 2005), which highlighted three categories of containment loss for stationary vessels, consisting of continuous release from the 10 mm-diameter hole, continuous release in 10 min at constant release, and instantaneous release.

Three threat zones result from the consequences of the toxicity, VCE, and jet fire scenarios, which are red, orange, and yellow. Only the number of employees in the red zone of the process plant area is considered when calculating the mortality rate, on the basis of the ratio of the area of the red zone to the area of the process plant area (Gaikwad et al., 2016). In the present case study, the process area section had an area of 300,562 ft^2^, whereas other sections, such as the workshop, utilities, and administration building, were not included in the mortality rate calculation.

The location of the MeOH plant was chosen in Manjung, Perak, which was used for all operating pressure variations in this study. The plant building at this site was assumed to be an unprotected two-story building with air exchange rates of 0.29 and 0.23 per hour during day and night conditions, respectively. Atmospheric data were also collected at the site, including air temperature, wind direction probability during the year, wind speed during day and night, and humidity, as tabulated in Table 2.

**Table 2:** Manjung methanol plant's site and atmospheric data.

Site		
Location	Manjung, Perak, Malaysia	
Building air exchange per hour (unsheltered double storied) and time	Day	0.29 and 3 pm.
	Night	0.23 and 11 pm.
Atmospheric Data		
Wind speed and stability class	Day	2.23 m/s and stability class B
	Night	1.03 m/s and stability class B
Average air temperature	29 °C	
Inversion	No	
Humidity	84%	

### 2.2 ANFIS prediction method

Four chemicals have been identified as components of the gas mixture emitted from the high-pressure reactor tank: MeOH and CO as products and excess H_2_ and CO_2_ as unreacted raw material. H_2_ and MeOH could lead to toxicity, flash fire, VCE, and jet fire events, whereas CO and CO_2_ only lead to toxicity events. H_2_ has a red-zone threat effect for VCE and jet fire events. MeOH leads to toxic and jet fire events. CO and CO_2_ contribute to toxicity. As the plant was modified with different pressure conditions between 76 and 500 bar, the resulting volume and mass in the tank differed at different pressures, resulting in different fatality rates in the red threat zone. Different sizes of leaks were used as follows: 10 (small), 25 (medium), and 160 mm (large). Day and night conditions were indicated by different wind speed and stability classes, which contribute to the different areas affected and the distance of the red threat zone. For the toxicity events of MeOH, CO, and CO_2_, wind direction was considered as another input variable. The inputs of the plant included the reactor pressure at reaction condition, mass and volume of the chemicals, wind speed, leak size, and wind direction selected as inputs to ANFIS. Meanwhile, all other inputs in ALOHA were assumed to be constant. The evaluation of the chemicals and the events that occurred are summarized in Table 3.

**Table 3:** Data for simulation prediction using ANFIS.

Item No	Input	Chemical	Event	Output prediction	Prediction simulation
1	1 Pressure (bar) 2 Mass (kg) 3 Volume (m3) 4 Leakage size (mm) 5 Wind Speed (m/s	MeOH	Jet fire	Fatality rate (60 data)	Simulation 1: ANFIS using trimf for fatality rate of MeOH jet fire Simulation 2: ANFIS using gaussmf for fatlity rate of MeOH jet fire
2		H2	Jet fire	Fatality rate (60 data)	Simulation 3: ANFIS using trimf for fatality rate of H2 jet fire Simulation 4: ANFIS using gaussmf for fatality rate of H2 jet fire
3	1 Pressure (bar) 2 Mass (kg) 3 Volume (m3) 4 Leakage size (mm) 5 Wind Speed (m/s) 6 Wind direction (o)	H2	Vapor cloud explosion (VCE)	Fatality rate (960 data)	Simulation 5: ANFIS using trimf for fatality rate of H2 VCE Simulation 6: ANFIS using gaussmf for fatality rate of H2 VCE
4		MeOH	Toxicity	Fatality rate (960 data)	Simulation 7: ANFIS using trimf for fatality rate of MeOH toxicity Simulation 8: ANFIS using gaussmf for fatality rate of MeOH toxicity
5		CO2	Toxicity	Fatality rate (480 data)	Simulation 9: ANFIS using trimf for fatality rate of CO2 toxicity Simulation 10: ANFIS using gaussmf for fatality rate of CO2 toxicity
6		CO	Toxicity	Fatality rate (960 data)	Simulation 11: ANFIS using trimf for fatality rate of CO toxicity Simulation 12: ANFIS using gaussmf for fatality rate of CO toxicity

#### 2.2.1 ANFIS algorithm

As shown in Table 3, five and six input data from a simulation of the consequences method for high-pressure MeOH production using HYSYS, ALOHA, and MARPLOT software were used to perform ANFIS prediction. The five-input data included five parameters, namely, pressure, mass, volume, wind speed, and size of leakage. The output data were fatality rate in the red zone threat. These five input data were simulated for the mortality rate of MeOH and H_2_ jet fire. The six input data were the same as the five input data, with the addition of wind direction, and the output data were the fatality rate in the red zone threat. These six input data were simulated for the area affected by MeOH toxicity, CO_2_ toxicity, CO toxicity, and H_2_ VCE. The ANFIS analysis was simulated in MATLAB version 2016b by using the ANFIS toolbox. Figure 2 shows the order in which the ANFIS analysis was performed.

ANFIS data were simulated using the gradient descent method, which 70% data for training, 15% data for validation and 15% data for testing; and output prediction was conducted using the least squares method. The ANFIS prediction method consists of four steps. First, the input data and the output from the consequences method are uploaded to the ANFIS toolbox. The second step is to assign the membership function (MF) by computing the fuzzy inference system (FIS). The third step is training and collecting data errors. The last step is predicting the output within the range of simulation output data. In this study, two shapes were simulated, namely, triangular MF (trimf) and Gaussian MF (gaussmf). Two metrics were also used to evaluate the performance of the ANFIS prediction method compared with the consequence prediction method using ALOHA and MARPLOT. These are the standard error of regression (S) and the symmetric mean absolute percentage error (SMAPE). S is defined as the sum of the differences between the result of the consequence prediction method ($Y_{i}^{cons}$) and the result of the ANFIS prediction method ($Y_{i}^{ANFIS}$), where the best value was obtained when S is closer to 0 (Frost, 2019). The equation for S is shown in Eq. (1):

(1) $$S=\sqrt{\frac{\sum_{i=1}^{N}{\left(Y_{i}^{\text{cons}}-Y_{i}^{\text{ANFIS}}\right)}^{2}}{N-2}}$$

SMAPE is defined as the percent error between simulation data and predicted data, where the target is to keep the percent error as small as possible, i.e., close to 0 (Makridakis, 1993; Tofallis, 2015). A value of 0.3 or 30% error is considered acceptable to perform the simulation or the predicted model (Darnaculleta, 2020). Eq. (2) could be used for the percentage error via SMAPE as follows:

(2) $$SMAPE=\frac{\sum_{i=1}^{N}\left|Y_{i}^{\text{cons}}-Y_{i}^{\text{ANFIS}}\right|}{\sum_{i=1}^{N}\left(Y_{i}^{\text{cons}}+Y_{i}^{\text{ANFIS}}\right)}\times 100\%$$

where N is the amount of data.

**Figure 3:**
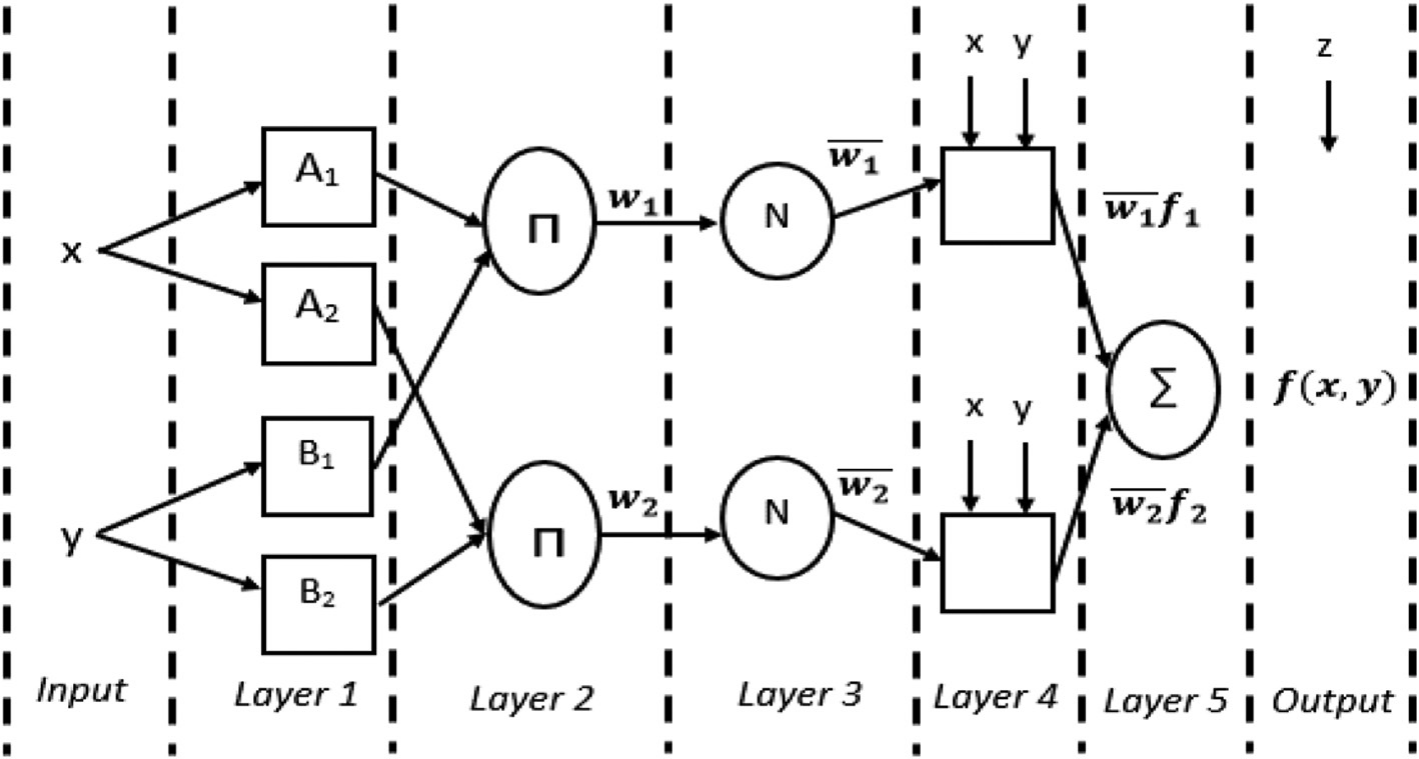
ANFIS model architecture.

The ANFIS algorithm combines an artificial neural network (ANN) with a Takagi-Sugeno fuzzy type inference system. It is built from a set of rules where each predicted output rule is the result of linear inputs multiplied by constant. The five layers developed in the ANFIS architecture are the fuzzification layer or the first layer, the second layer as the product layer, the normalized layer as the third layer, the defuzzification layer as the fourth layer, and the output layer as the fifth layer, as shown in Figure 3. In the fuzzification layer, the input x_i_ for the membership function is assigned in accordance with the specified range of this input. The second layer consists of the weighting function, w_i_, resulting from the combination of the different inputs in the first layer. In addition, the normalized layer consists of all fixed nodes, n, where the product takes the form of a weighting function, $\bar{w_{i}}$. In the fourth layer, defuzzification produces a product $\bar{w_{i}}f_{i}\text{,}$ where $f_{i}$ consists of linear parameters of p_i_, q_i_ and r_i_ and is defined as $f_{i}=\left(p_{i}x_{i}+q_{i}x_{i}+r_{i}\right),i=1,2$ (Kasmuri et al., 2019).

#### 2.2.2 Comparison of input data for consequence and ANFIS prediction

Table 1 shows that the consequence prediction models required 24 inputs data for toxic, flash fire, and jet fire incidents and 26 inputs for the VCE incident. With the ANFIS prediction method, only five inputs were required to predict the fatality rate for H_2_ and MeOH jet fires, while six inputs were required to estimate the fatality rate for H_2_ VCE, MeOH, CO, and CO_2_ toxicity incidents (Table 3). This comparison showed that the ANFIS prediction method simplified the consequence prediction method in terms of the inputs required to estimate the severity parameter, which is the fatality rate in the red zone.

## 3 Results and discussion

### 3.1 Consequence prediction analysis

Sixty simulations were performed for MeOH and H_2_ jet incidents, each of which yielded 60 fatality rate data. The variation in reactor pressure resulted in variation in volumes and masses of MeOH and H_2_ in the output mixture. For MeOH and H_2_, the data for pressure ranged from 76 bar to 500 bar. For MeOH, the resulting volume ranged from 0.39 m^3^ to 4.61 m^3^, while the resulting mass ranged from 16.7 kg to 1990 kg. For H_2_, the resulting volume ranged from 0.57 m^3^ to 4.27 m^3^, while the resulting mass ranged between 16.2 and 42.3 kg. The data for wind speed varied for two values: 1.03 m/s for nighttime and stability class F and 2.23 m/s for daytime with stability class B. The data for the size of the leak were 10 mm leak, 25 mm leak, and 160 mm rupture. The resulting fatality rate ranged from 0% to 12.91% for MeOH jet fire and from 0% to 2.1% for H_2_ jet fire.

A total of 960 output data of fatality rate were identified for H_2_ VCE, MeOH toxicity, CO toxic incidents and 480 output data of fatality rate for CO_2_ toxicity. Different operating pressures from 76 bar to 500 bar of the reactor resulted in different volumes and masses of H_2_, MeOH, CO_2_, and CO in the mixture product. For H_2_, the resulting volume ranged from 0.57 m^3^ to 4.27 m^3^, while the resulting masses ranged from 16.2 kg to 42.3 kg. For MeOH, the resulting volume ranged from 0.39 m^3^ to 4.61 m^3^, while the resulting mass ranged from 16.7 kg to 1990 kg. For CO_2_, the resulting volume ranged from 0.34 m^3^ to 2.35 m^3^, while the resulting mass ranged from 105.3 kg to 297 kg. For CO, the resulting volume ranged from 0.01 m^3^ to 0.27 m^3^, while the resulting mass ranged between 3 and 13.7 kg. The data for wind speed varied for two values: 1.03 m/s for nighttime and stability class F and 2.23 m/s for daytime with stability class B. The data for the size of the leakage were 10 mm leak, 25 mm leak, and 160 mm rupture, while the data for the wind direction were between 0° and 337.5°. The resulting fatality rate ranged from 0% to 6.98% For H_2_ VCE, it was 0%–3.67% for MeOH toxicity. Meanwhile, the resulting fatality rate ranged from 0% to 19.73% for CO_2_ toxicity and from 1.47% to 9.16% for CO toxicity.

All the incidents resulting from the consequence prediction method simulated in HYSYS, ALOHA, and MARPLOT are summarized in Table 4, which shows the range of input data consisting of the operating pressure, volume of chemicals, mass of chemicals, size of the leakage, wind speed, and wind direction and the output data, i.e., the fatality rate in the red zone threat. All these input and output data were then exported to the ANFIS toolbox in MATLAB for training, and the predicted output for the fatality rates was determined.

**Table 4:** Summary range of input and output data for each incident.

Incident	Number of run	Range of pressure (bar)	Range of volume (m^3^)	Range of mass (kg)	Range of leakage size (mm)	Wind speed (m/s)	Wind direction (^o^)	Percentage area affected in red zone (%)
Methanol jet fire	60	76.4–500	0.39–4.61	16.7–1990	10–160	1.03–2.23		0–12.91
Hydrogen jet fire	60	76.4–500	0.57–4.27	16.2–42.3	10–160	1.03–2.23		0–2.1
Hydrogen VCE	960	76.4–500	0.57–4.27	16.2–42.3	10–160	1.03–2.23	0–337.5	0–6.98
Methanol toxic	960	76.4–500	0.39–4.61	16.7–1990	10–160	1.03–2.23	0–337.5	0–3.67
Carbon dioxide toxic	480	76.4–500	0.34–2.35	105.3–297	10–160	1.03	0–337.5	0–19.73
Carbon monoxide toxic	960	76.4–500	0.01–0.27	3–13.7	10–160	1.03–2.23	0–337.5	1.47–9.16

### 3.2 ANFIS prediction analysis

The fatality rate results from the ANFIS trimf and ANFIS gaussmf prediction methods compared with those from consequence prediction method for MeOH jet fire, H_2_ jet fire, MeOH toxicity, H_2_ VCE, CO_2_ toxicity, and CO toxicity consequences were validated using the standard error of the regression S and percent error measure. For MeOH jet fire, the S and percent error results were nearly similar for both ANFIS methods. ANFIS gaussmf exhibited the value closest to 0 for both measurements, and both methods exhibited a percent error below 30%, indicating that the results are acceptable. These results showed that ANFIS gaussmf is the best prediction method for the fatality rate of MeOH jet incidents. For H_2_ jet fire, the results of S and percent error were also nearly the same for both ANFIS methods. ANFIS trimf was closest to 0 for both measurements, and both methods showed an acceptable percent error. These results showed that ANFIS trimf is the best prediction method for the fatality rate of H_2_ jet fire incidents.

For H_2_ VCE, the results of S and the percent error were almost similar for both ANFIS methods, that is, 0.0088 and 0.0093 for trimf and gaussmf, respectively. The ANFIS trimf value was closest to 0 for both measurements, and both methods had a valid percent error, indicating that the results are acceptable. These results showed that ANFIS trimf is the best prediction method for the fatality rate of H_2_ VCE incidents. For MeOH toxicity, the results of S and percent error differed for both ANFIS methods at 0.089 and 0.099 for trimf and gaussmf, respectively. ANFIS trimf produced a percent error of 2.17%, while ANFIS gaussmf produced an error of 3.43%. Both methods still had valid percent errors, proving that the results are acceptable. These results showed that ANFIS trimf is the best prediction method for fatality rate of MeOH toxicity incidents.

**Figure 4:**
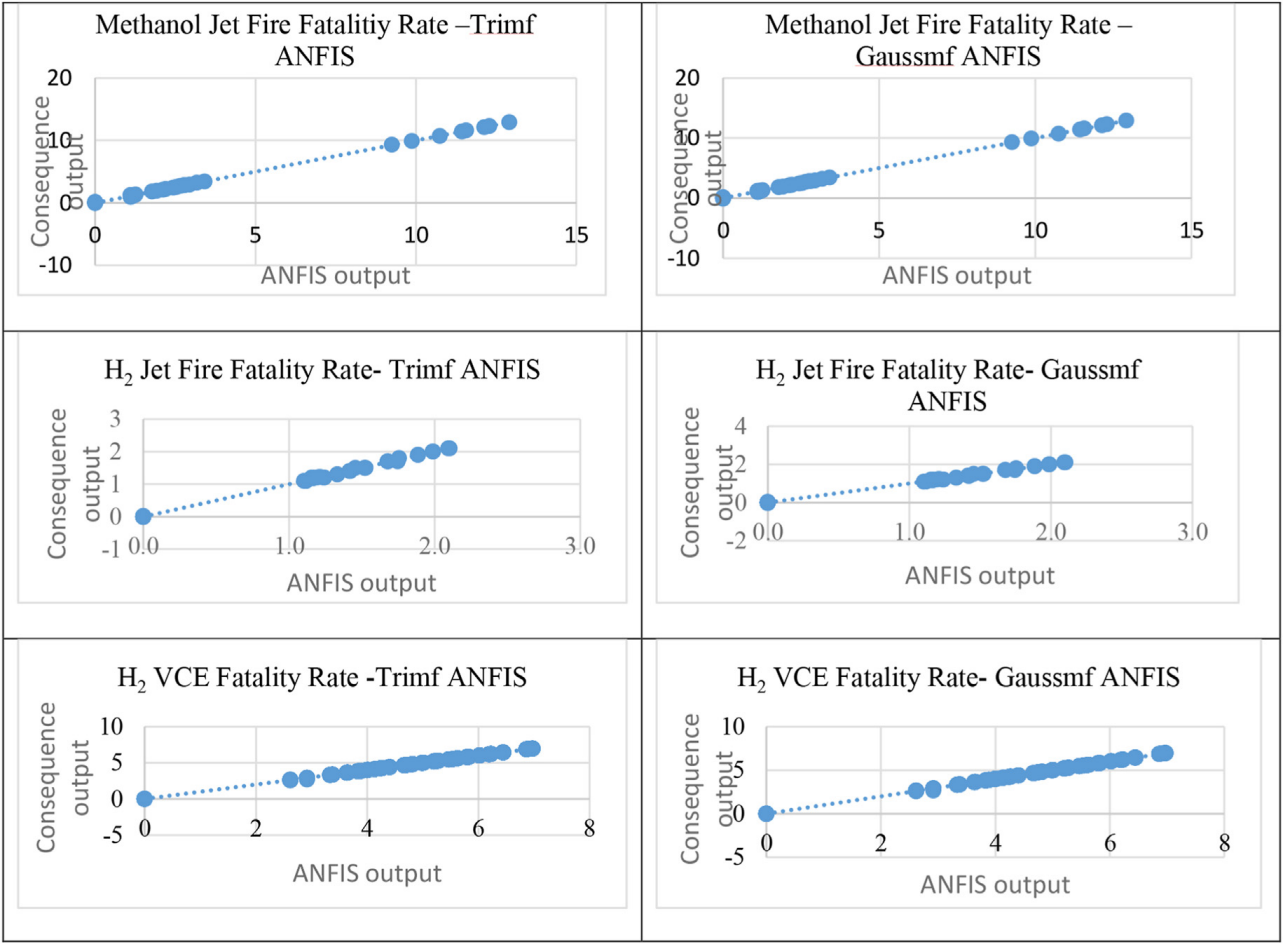
Regression line for methanol jet fire, H_2_ jet fire and H_2_ VCE.

**Figure 5:**
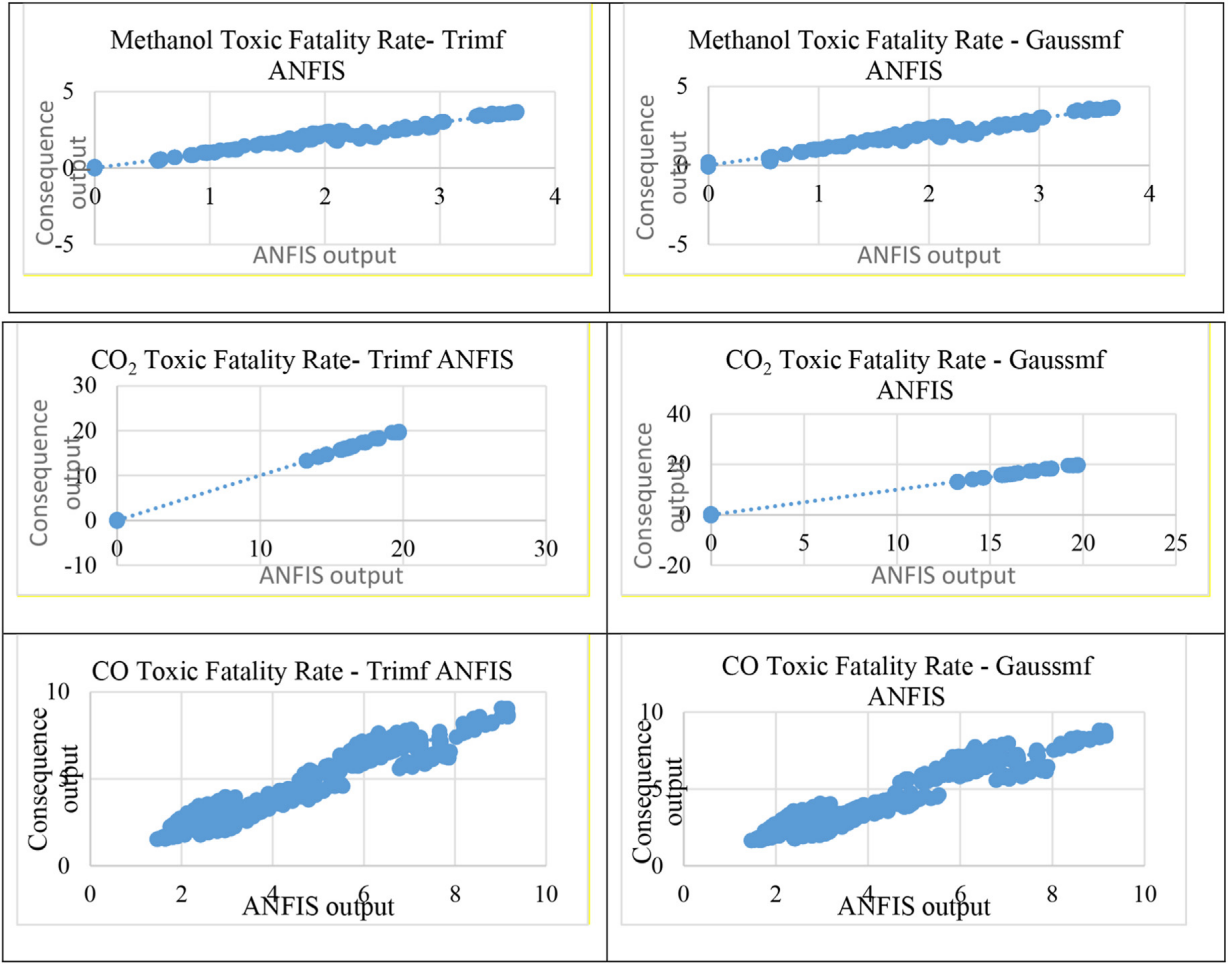
Regression line for methanol, CO and CO_2_ toxicity.

For CO_2_ toxicity, the regression S results gave 0.049 and 0.112 for trimf and gaussmf, respectively, while the percent error was 0.23% for trimf and 0.68% for gaussmf. These results showed that ANFIS trimf is the best prediction method for fatality rate of CO_2_ toxicity incident. The results of CO showed S values of 0.571 for trimf and 0.533 for gaussmf. As for the percentage error, the value of trimf was 4.74%, which is close to 0, while the gaussmf value was 5%. Therefore, ANFIS trimf is the best prediction method for fatality rate of CO toxicity incidents. All of the regression line results of ANFIS prediction versus consequence prediction for MeOH jet fire, H_2_ jet fire, and H_2_ VCE are shown in Figure 4, and Figure 5 shows the regression line of ANFIS prediction versus consequence prediction for MeOH, CO_2_, and CO toxicities. Table 5 summarizes all regression S and percent error results by using ANFIS trimf and gaussmf for all MeOH jet fire, H_2_ jet fire, MeOH toxicity, H_2_ VCE, CO_2_ toxicity, and CO toxicity incidents.

**Table 5:** Regression and percent error of ANFIS prediction.

Method	Standard error of regression, S	Percentage error (%)
MeOH jet fire		
ANFIS method using trimf	0.050	0.69
ANFIS method using gaussmf	0.045	0.67
H_2_ jet fire		
ANFIS method using trimf	0.019	0.88
ANFIS method using gaussmf	0.021	1.12
H_2_ VCE		
ANFIS method using trimf	0.0088	0.02
ANFIS method using gaussmf	0.0093	0.04
MeOH toxicity		
ANFIS method using trimf	0.089	2.17
ANFIS method using gaussmf	0.099	3.43
CO_2_ toxicity		
ANFIS method using trimf	0.049	0.23
ANFIS method using gaussmf	0.112	0.68
CO toxicity		
ANFIS method using trimf	0.571	4.74
ANFIS method using gaussmf	0.533	5.00

### 3.3 Comparison between consequence and ANFIS prediction analysis

A comparison was performed between the consequence prediction method and the ANFIS prediction method in terms of the red zone fatality rate. The accuracy of the ANFIS method against the results of the consequence method was measured by the percentage error value. The ANFIS prediction method using the triangle MF provided the lowest percentage error at 0.02% for the H_2_ VCE incident, while the highest percentage at 5% was provided by the ANFIS prediction method using gaussmf for the CO toxicity incident. The best method was selected on the basis of the lowest percentage error, where ANFIS triangular MF was found to be the best method for five incidents. Meanwhile, ANFIS gaussian MF was determined to be the best method for one incident. The results for the whole incident proved that ANFIS triangular MF is a better performance prediction method than ANFIS gaussian MF. Table 6 illustrates the fatality rate output data for consequence prediction and ANFIS prediction methods, and Table 7 shows the best prediction method for each incident.

**Table 6:** Output data for the consequence and ANFIS prediction methods for fatality rate.

Incident	Range of pressure (bar)	Range of volume (m^3^)	Range of mass (kg)	Range of leakage size (mm)	Range of wind speed (m/s)	Range of wind direction (^o^)	Range of fatality rate (%) – Consequence method	Range of fatality rate (%) –ANFIS trimf	Range of fatality rate (%) – ANFIS gaussmf
MeOH jet fire	76.4–500	0.39–4.61	16.7–1990	10–160	1.03–2.23		0–12.91	−0.03–12.9	−0.08–12.9
							% Error	0.69%	0.67%
H_2_ jet fire	76.4–500	0.57–4.27	16.2–42.3	10–160	1.03–2.23		0–2.1	−0.01–2.1	−0.03–2.1
							% Error	0.88%	1.12%
H_2_ VCE	76.4–500	0.57–4.27	16.2–42.3	10–160	1.03–2.23	0–337.5	0–6.98	−0.001–6.97	−0.009–6.97
							% Error	0.02%	0.04%
MeOH toxicity	76.4–500	0.39–4.61	16.7–1990	10–160	1.03–2.23	0–337.5	0–3.67	−0.04–3.69	−0.09–3.68
							% Error	2.17%	3.43%
CO_2_ toxicity	76.4–500	0.34–2.35	105.3–297	10–160	1.03	0–337.5	0–19.73	−0.15–19.78	−0.29–19.78
							% Error	0.23%	0.68%
CO toxicity	76.4–500	0.01–0.27	3–13.7	10–160	1.03–2.23	0–337.5	1.47–9.16	1.53–9.06	1.65–8.81
							% Error	4.74%	5.00%

**Table 7:** Best ANFIS prediction method for each incident.

Incident	Best Prediction Method
MeOH jet fire	ANFIS gaussian MF
H_2_ jet fire	ANFIS triangular MF
H_2_ VCE	ANFIS triangular MF
MeOH toxicity	ANFIS triangular MF
CO_2_ toxicity	ANFIS triangular MF
CO toxicity	ANFIS triangular MF

## 4 Conclusion

The fatality rates for MeOH plants with six incident scenarios were predicted using the consequence prediction method and the ANFIS prediction method. The performance of ANFIS prediction gave the best fit of 0.0088, with the lowest percent error of 0.02% for the H_2_ VCE incident. For the other incidents, the regression and percent error did not exceed 0.571 and 5%, respectively. Thus, prediction using the ANFIS method proved to be the simpler and alternative method for predicting the fatality rate, which is equivalent to the consequence prediction method. Although a low percentage error was achieved, further studies for different input pressures, such as 125, 175, and 225 bar, are recommended to test the ability of ANFIS to predict.

## Declarations

### Author contribution statement

Mohd Aizad Ahmad; Zulkifli abdul rashid: Conceived and designed the experiments; Performed the experiments; Analyzed and interpreted the data; Contributed reagents, materials, analysis tools or data; Wrote the paper.

Ateyah Awad Alzahrani; Mohanad El-Harbawi: Analyzed and interpreted the data; Contributed reagents, materials, analysis tools or data; Wrote the paper.

### Funding statement

Assoc Prof Ir Dr zulkifli abdul rashid was supported by the School of Chemical Engineering, College of Engineering, Universiti Teknologi MARA (UiTM), Ministry of Higher Education, Malaysia [Ministry of Higher Education (MOHE) under 600-RMC/FRGS 5/3 (168/2021) and 600-RMC/SRC/5/3 (010/2020)]. Mohanad El-Harbawi was supported by the Deputyship for Research & Innovation, Ministry of Education in Saudi Arabia through the project no. (IFKSURG-476).

### Data availability statement

Data will be made available on request.

### Declaration of interests statement

The authors declare no conflict of interest.

### Additional information

No additional information is available for this paper.
